# Supplementary material for: Oligodendrocytes and myelin limit neuronal plasticity in visual cortex
Source: Nature. 2024 Aug 21;633(8031):856–63. doi: 10.1038/s41586-024-07853-8 (PMC11424474; doi:10.1038/s41586-024-07853-8)
Supplement: Supplementary file 1 — Supplementary Table 1 contains additional statistical details; Supplementary Table 2 contains additional antibody information. [file 41586_2024_7853_MOESM1_ESM.pdf]

nature portfolio

https://doi.org/10.1038/s41586-024-07853-8

Supplementary information

Oligodendrocytes and myelin limit neuronal plasticity in visual cortex

In the format provided by the authors and unedited

Supplementary Tables

Table S1 | Statistical analysis

Figure	Sample size	Statistical test	Values
1h	8 mice	Paired t-test (two-tailed)	t = 5.494, p = 0.0009
1i	8 mice	Paired t-test (two-tailed)	t = 3.06, p = 0.0183
1j	8 mice	Paired t-test (two-tailed)	t = 0.3199, p = 0.7584
1k	8 mice	Paired t-test (two-tailed)	t = 3.84, p = 0.0064
1l	5 mice	Paired t-test (two-tailed)	t = 3.867, p = 0.018
1m	5 mice	Paired t-test (two-tailed)	t = 1.64, p = 0.1763
2b	3-6 mice per age, per genotype	Two-way ANOVA followed by Sidak's multiple comparisons test	Genotype:<br>F = 165.9, p < 0.0001<br>Interaction:<br>F = 9.55, p = 0.0002<br><br>CTL – cKO<br>P28: p = 0.1808<br>P35: p < 0.0001<br>P45: p < 0.0001<br>P60: p < 0.0001
2c	3-4 mice per age, per genotype	Two-way ANOVA followed by Sidak's multiple comparisons test	Genotype:<br>F = 0.7504, p = 0.7504<br>Interaction:<br>F = 0.0979, p = 0.7608<br><br>CTL vs cKO<br>P28: p > 0.9999<br>P60: p = 0.8672
2i	8-9 mice per genotype	Two-way ANOVA followed by Sidak's multiple comparisons test	Genotype:<br>F = 30.64, p < 0.0001<br>Interaction:<br>F = 0.4264, p = 0.5237
2j	8 mice	Two one-way repeated measures ANOVAs (one for contra and one for ipsi) followed by Tukey's multiple comparisons test	Contra<br>F = 1.733, p = 0.1804<br>Baseline vs MD4: p = 0.9863<br>Baseline vs MD8: p = 0.1922<br>MD4 vs MD8: p = 0.1814<br><br>Ipsi<br>F = 30.17, p < 0.0001<br>Baseline vs MD4: p = 0.5364<br>Baseline vs MD8: p = 0.0004<br>MD4 vs MD8: p = 0.0015
2k	9 mice	Two one-way repeated measures ANOVAs (one for contra and one for ipsi) followed by Tukey's multiple comparisons test	Contra<br>F = 66.55, p < 0.0001<br>Baseline vs MD4: p = 0.0002<br>Baseline vs MD8: p < 0.0001<br>MD4 vs MD8: p = 0.0015<br><br>Ipsi<br>F = 20.66, p < 0.0001<br>Baseline vs MD4: p = 0.0575<br>Baseline vs MD8: p = 0.0013<br>MD4 vs MD8: p = 0.0053
2l	8-9 mice per genotype	Unpaired t-test (two-tailed)	t = 5.082, p = 0.0001
2m	8-9 mice per genotype	Two-way ANOVA followed by Sidak's multiple comparisons test	Genotype:<br>F = 36.22, p < 0.0001<br>Interaction:<br>F = 15.62, p < 0.0001<br><br>CTL vs cKO<br>MD 4d: p = 0.0003<br>MD 8d: p = 0.0007<br>Rec 4d: p = 0.0002<br>Rec 8d: p = 0.0013

2n	8-9 mice per genotype	Two-way ANOVA followed by Sidak's multiple comparisons test	Genotype:<br>F = 1.247, p = 0.3008<br>Interaction:<br>F = 1.922, p = 0.1859<br><br>CTL vs cKO<br>MD 4d: p = 0.5115<br>MD 8d: p = 0.9725<br>Rec 4d: p = 0.2595<br>Rec 8d: p = 0.9961
3d	10 control mice (111 dendrites)<br><br>10 cKO mice (97 dendrites)	Two-way repeated measures ANOVA followed by Holm-Sidak's multiple comparisons test	Genotype:<br>F = 7.394, p = 0.0071<br>Interaction:<br>F = 2.115, p = 0.0972<br><br>CTL vs cKO<br>Day -2: p = 0.0311<br>Day 0: p = 0.0103<br>Day 2: p = 0.0095<br>Day 4: p = 0.0391
3e	10 control mice (111 dendrites)<br><br>10 cKO mice (97 dendrites)	Mixed-effects analysis (REML) followed by Holm-Sidak's multiple comparisons test	Genotype:<br>F = 8.82, p = 0.0033<br>Interaction:<br>F = 0.6344, p = 0.5931<br><br>CTL vs cKO<br>Day -2: p = 0.3037<br>Day 0: p = 0.0103<br>Day 2: p = 0.0178<br>Day 4: p = 0.0586
3f	10 control mice (111 dendrites)<br><br>10 cKO mice (97 dendrites)	Mixed-effects analysis (REML) followed by Holm-Sidak's multiple comparisons test	Genotype:<br>F = 13.9, p = 0.0002<br>Interaction:<br>F = 1.101, p = 0.3484<br><br>CTL vs cKO<br>Day -2: p = 0.0288<br>Day 0: p = 0.0048<br>Day 2: p = 0.0521<br>Day 4: p = 0.192
3g	10 control mice (111 dendrites)<br><br>10 cKO mice (97 dendrites)	Mixed-effects analysis (REML) followed by Holm-Sidak's multiple comparisons test	Genotype:<br>F = 3.483, p = 0.0634<br>Interaction:<br>F = 0.9613, p = 0.4107<br><br>CTL vs cKO<br>Day -2: p = 0.7148<br>Day 0: p = 0.2422<br>Day 2: p = 0.1721<br>Day 4: p = 0.1721
3h	10 control mice (111 dendrites)<br><br>10 cKO mice (97 dendrites)	Two-way repeated measures ANOVA followed by Holm-Sidak's multiple comparisons test	Genotype:<br>F = 2.471, p = 0.1175<br>Interaction:<br>F = 2.64, p = 0.0486<br><br>CTL vs cKO<br>Day -2: p = 0.7333<br>Day 0: p = 0.7241<br>Day 2: p = 0.9743<br>Day 4: p = 0.0110
4c	10 control mice (110 dendrites)<br><br>10 cKO mice (96 dendrites)	Unpaired t-test (two-tailed)	t = 0.3287, p = 0.7427
4d	10 control mice (110 dendrites)	Unpaired t-test (two-tailed)	t = 2.826, p = 0.0052

	10 cKO mice (96 dendrites)		
4e	10 control mice (110 dendrites)<br><br>10 cKO mice (96 dendrites)	Unpaired t-test (two-tailed)	t = 2.56, p = 0.0112
4f	10 control mice (110 dendrites)<br><br>10 cKO mice (96 dendrites)	Unpaired t-test (two-tailed)	t = 1.627, p = 0.1052
4g	10 control mice (996 spine pairs)<br>10 cKO mice (704 spine pairs)	Monte Carlo p value calculated by summing the tail of the histogram from 10000 shuffled spine pairings	p = 0.686
4h	10 control mice (996 spine pairs)<br>10 cKO mice (704 spine pairs)	Monte Carlo p value calculated by summing the tail of the histogram from 10000 shuffled spine pairings	p = 0.939
4i	10 control mice (996 spine pairs)<br>10 cKO mice (704 spine pairs)	Monte Carlo p value calculated by summing the tail of the histogram from 10000 shuffled spine pairings	p = 0.913
4j	10 control mice (996 spine pairs)<br>10 cKO mice (704 spine pairs)	Monte Carlo p value calculated by summing the tail of the histogram from 10000 shuffled spine pairings	p = 0.66
4k	10 control mice (996 spine pairs)<br>10 cKO mice (704 spine pairs)	Monte Carlo p value calculated by summing the tail of the histogram from 10000 shuffled spine pairings	p = 0.862
4l	10 control mice (996 spine pairs)<br>10 cKO mice (704 spine pairs)	Monte Carlo p value calculated by summing the tail of the histogram from 10000 shuffled spine pairings	p = 0.001
4m	10 control mice (996 spine pairs)<br>10 cKO mice (704 spine pairs)	Monte Carlo p value calculated by summing the tail of the histogram from 10000 shuffled spine pairings	p = 0.002
4n	10 control mice (996 spine pairs)<br>10 cKO mice (704 spine pairs)	Monte Carlo p value calculated by summing the tail of the histogram from 10000 shuffled spine pairings	p = 0.022
5b	3 mice per genotype	Unpaired t-test (two-tailed)	t = 1.331, p = 0.2538
5c	3 mice per genotype	Unpaired t-test (two-tailed)	t = 1.635, p = 0.1773
5d	3 mice per genotype	Unpaired t-test (two-tailed)	t = 0.3877, p = 0.718
5g	5 control mice (15 cells)<br>5 cKO mice (14 cells)	Unpaired t-test (two-tailed)	t = 4.195, p = 0.0003
5h	5 control mice (15 cells)<br>5 cKO mice (14 cells)	Unpaired t-test (two-tailed)	t = 0.4712, p = 0.6413
S1b	8 mice	Paired t-test (two-tailed)	t = 2.565, p = 0.0373
S1c	8 mice	Paired t-test (two-tailed)	t = 2.176, p = 0.066
S1f	5 mice	Paired t-test (two-tailed)	t = 3.637, p = 0.022
S3b	4-6 mice per genotype	Unpaired t-test (two-tailed)	t = 0.5169, p = 0.6237
S3c	4-6 mice per genotype	Unpaired t-test (two-tailed)	t = 2.653, p = 0.0291
S3d	4-6 mice per genotype	Unpaired t-test (two-tailed)	t = 3.995, p = 0.004
S3e	4-6 mice per genotype	Unpaired t-test (two-tailed)	t = 8.612, p = 0.0001
S4c	4-6 mice per genotype	Unpaired t-test (two-tailed)	t = 5.828, p = 0.0004
S4d	4-6 mice per genotype	Unpaired t-test (two-tailed)	t = 7.076, p = 0.0001
S5b	4-6 mice per genotype	Unpaired t-test (two-tailed)	t = 0.1984, p = 0.8477
S5d	4-5 mice per genotype	Unpaired t-test (two-tailed)	t = 3.461, p = 0.0105
S5f	4-6 mice per genotype	Unpaired t-test (two-tailed)	t = 2.982, p = 0.0176
S5g	4-6 mice per genotype	Unpaired t-test (two-tailed)	t = 2.195, p = 0.0594
S6b	4 mice per genotype	Welch’s t-test	t = 1.718, p = 0.1748
S6c	4 mice per genotype	Unpaired t-test (two-tailed)	t = 0.3735, p = 0.7216
S6g	4 mice per genotype	Unpaired t-test (two-tailed)	t = 0.595, p = 0.5736

S6h	4 mice per genotype	Unpaired t-test (two-tailed)	t = 0.4586, p = 0.6627
S8b	8-9 mice per genotype	Two-way ANOVA followed by Sidak's multiple comparisons test	Genotype:<br>F = 55.3, p < 0.0001<br>Interaction:<br>F = 11.73, p < 0.0001<br><br>CTL vs cKO<br>Base: p = 0.0041<br>MD 4d: p < 0.0001<br>MD 8d: p = 0.0004<br>Rec 4d: p < 0.0001<br>Rec 8d: p = 0.0002
S8c	8-9 mice per genotype	Two-way ANOVA followed by Sidak's multiple comparisons test	Genotype:<br>F = 42.31, p < 0.0001<br>Interaction:<br>F = 2.183, p = 0.0816<br><br>CTL vs cKO<br>Base: p = 0.0002<br>MD 4d: p = 0.0022<br>MD 8d: p = 0.0008<br>Rec 4d: p = 0.0009<br>Rec 8d: p = 0.0011
S9b	10 control mice (3484 spines)<br>10 cKO mice (2438 spines)	Kolmogorov-Smirnov test	p < 0.0001
S9c	10 control mice (3809 spines)	Pearson r correlation	r = 0.4641, p < 0.0001
S9d	10 cKO mice (3484 spines)	Pearson r correlation	r = 0.5043, p < 0.0001
S9e	10 control mice (996 spine pairs)	Pearson r correlation	r = 0.02819, p = 0.3742
S9f	10 cKO mice (704 spine pairs)	Pearson r correlation	r = 0.1692, p < 0.0001
S9g,h	N/A	Example of one shuffled spine pairing as part of Monte Carlo simulation; no statistics involved	N/A
S10b	5 control mice (15 cells)<br>5 cKO mice (14 cells)	Unpaired t-test (two-tailed)	t = 3.563, p = 0.0014
S10c	5 control mice (15 cells)<br>5 cKO mice (14 cells)	Unpaired t-test (two-tailed)	t = 0.1121, p = 0.9116
S10d	5 control mice (15 cells)<br>5 cKO mice (14 cells)	Unpaired t-test (two-tailed)	t = 2.029, p = 0.0525
S10e	5 control mice (15 cells)<br>5 cKO mice (14 cells)	Unpaired t-test (two-tailed)	t = 1.598, p = 0.1218

Table S2 | Antibodies

Primary

Antibody	*Source*	*Identifier*	*Concentration*
Rabbit anti-ASPA	GeneTex	Cat# GTX113389; RRID AB_2036283	1:1000
Chicken anti-GFP	Rockland	Cat# 600-901-215; RRID AB_1537403	1:1000
Rat anti-MBP	Millipore	Cat# MAB386; RRID AB_94975	1:200
Rabbit anti-PDGFRα	W.B. Stallcup	N/A	1:200
Mouse anti-Bcas1	Santa Cruz	Cat# SC-136342; RRID AB_10839529	1:300
Rabbit anti-Caspr	Elior Peles	N/A	1:600
Rabbit anti-cleaved Caspase3	Cell Signaling	Cat# 9661S; RRID AB_2341188	1:200
Mouse anti-GFAP	Millipore	Cat# MAB360; RRID AB_11212597	1:1000
Goat anti-Sox9	R&D Systems	Cat# AF3075; RRID AB_2194160	1:2000
Rabbit anti-Iba1	Wako	Cat# 019-19741; RRID AB_839504	1:1000
Mouse anti-NF-L Degenotag	Encor	Cat# MCA-1D44; RRID AB_2923483	1:1000
Rabbit anti-NF-H	Abcam	Cat# ab8135; RRID AB_306298	1:1000
Mouse anti-PV	Swant	Cat# 235; RRID AB_10000343	1:1000
Biotinylated WFA	Vector Labs	Cat# B-1355; RRID AB_2336874	1:400

Secondary

Antibody	*Source*	*Identifier*	*Concentration*
Goat anti-rabbit AlexaFluor 488	Thermo Fisher Scientific	Cat# A-11034; RRID:AB_2576217	1:1000
Goat anti-rabbit AlexaFluor 594	Thermo Fisher Scientific	Cat# A-11012; RRID:AB_2534079	1:1000
Goat anti-rabbit AlexaFluor 647	Thermo Fisher Scientific	Cat # A-21245; RRID:AB_2535813	1:1000
Goat anti-chicken AlexaFluor 488	Thermo Fisher Scientific	Cat # A-11039; RRID:AB_142924	1:1000
Goat anti-rat AlexaFluor 647	Thermo Fisher Scientific	Cat# A-21247; RRID:AB_141778	1:1000
Goat anti-rat AlexaFluor 488	Jackson ImmunoResearch	Cat# 112-545-167; RRID:AB_2338362	1:1000
Goat anti-mouse AlexaFluor 488	Jackson ImmunoResearch	Cat# 115-545-166; RRID:AB_2338852	1:1000
Goat anti-mouse AlexaFluor 647	Thermo Fisher Scientific	Cat# A-21236; RRID:AB_2535805	1:1000
Goat anti-human AlexaFluor 594	Thermo Fisher Scientific	Cat# A-11014; RRID:AB_2534081	1:1000
Alexa Fluor® 594 Streptavidin	Jackson ImmunoResearch	Cat# 016-580-084; RRID:AB_2337250	1:1000
