# Supplementary material for: Oligodendrocytes and myelin limit neuronal plasticity in visual cortex
Source: Nature. 2024 Aug 21;633(8031):856–63. doi: 10.1038/s41586-024-07853-8 (PMC11424474; doi:10.1038/s41586-024-07853-8)
Supplement: Supplementary file 2 — Reporting Summary [file 41586_2024_7853_MOESM2_ESM.pdf]

nature portfolio

Corresponding author(s): Wendy Xin, Jonah Chan

Last updated by author(s): Jun 18, 2024

Reporting Summary

Nature Portfolio wishes to improve the reproducibility of the work that we publish. This form provides structure for consistency and transparency in reporting. For further information on Nature Portfolio policies, see our Editorial Policies and the Editorial Policy Checklist.

Statistics

For all statistical analyses, confirm that the following items are present in the figure legend, table legend, main text, or Methods section.

n/a	Confirmed	
☐	☒	The exact sample size (n) for each experimental group/condition, given as a discrete number and unit of measurement
☐	☒	A statement on whether measurements were taken from distinct samples or whether the same sample was measured repeatedly
☐	☒	The statistical test(s) used AND whether they are one- or two-sided<br>*Only common tests should be described solely by name; describe more complex techniques in the Methods section.*
☐	☒	A description of all covariates tested
☐	☒	A description of any assumptions or corrections, such as tests of normality and adjustment for multiple comparisons
☐	☒	A full description of the statistical parameters including central tendency (e.g. means) or other basic estimates (e.g. regression coefficient) AND variation (e.g. standard deviation) or associated estimates of uncertainty (e.g. confidence intervals)
☐	☒	For null hypothesis testing, the test statistic (e.g. F, t, r) with confidence intervals, effect sizes, degrees of freedom and P value noted<br>*Give P values as exact values whenever suitable.*
☐	☒	For Bayesian analysis, information on the choice of priors and Markov chain Monte Carlo settings
☒	☐	For hierarchical and complex designs, identification of the appropriate level for tests and full reporting of outcomes
☐	☒	Estimates of effect sizes (e.g. Cohen's d, Pearson's r), indicating how they were calculated

Our web collection on statistics for biologists contains articles on many of the points above.

Software and code

Policy information about availability of computer code

Data collection	In situ images were collected via proprietary Zeiss Zen 2 (blue edition, version 2.0.0.0) software. Intrinsic signal images were acquired using custom Linux software. In vivo images were acquired using ThorImage LS. Slice recordings were obtained with a Multiclamp 700B amplifier from Molecular Devices using WinWCP software.
Data analysis	Fluorescent images were analyzed using FIJI and MapManager software (https://mapmanager.net) written in Igor Pro (WaveMetrics) as previously described in Roth et al., Neuron, 2020. Intrinsic signal imaging data were analyzed using custom Matlab code as described in Cang et al., Visual Neuroscience, 2005. Slice recordings were analyzed using MiniAnalysis from Synaptosoft. Statistical analysis was performed using Matlab v2016b and Graphpad Prism v9.

For manuscripts utilizing custom algorithms or software that are central to the research but not yet described in published literature, software must be made available to editors and reviewers. We strongly encourage code deposition in a community repository (e.g. GitHub). See the Nature Portfolio guidelines for submitting code & software for further information.

Data

Policy information about availability of data

All manuscripts must include a data availability statement. This statement should provide the following information, where applicable:

- Accession codes, unique identifiers, or web links for publicly available datasets
- A description of any restrictions on data availability
- For clinical datasets or third party data, please ensure that the statement adheres to our policy

No databases were generated during the course of this study. Raw data for any main or supplemental figure can be supplied upon request.

Research involving human participants, their data, or biological material

Policy information about studies with human participants or human data. See also policy information about sex, gender (identity/presentation), and sexual orientation and race, ethnicity and racism.

Reporting on sex and gender	N/A
Reporting on race, ethnicity, or other socially relevant groupings	N/A
Population characteristics	N/A
Recruitment	N/A
Ethics oversight	N/A

Note that full information on the approval of the study protocol must also be provided in the manuscript.

Field-specific reporting

Please select the one below that is the best fit for your research. If you are not sure, read the appropriate sections before making your selection.

☒ Life sciences ☐ Behavioural & social sciences ☐ Ecological, evolutionary & environmental sciences

For a reference copy of the document with all sections, see nature.com/documents/nr-reporting-summary-flat.pdf

Life sciences study design

All studies must disclose on these points even when the disclosure is negative.

Sample size	We used published standards from comparable studies to determine sample size for all experiments (e.g. for histology experiments, see Mei et al., JNeurosci, 2016; for intrinsic signal imaging, see Kaneko et al., eLife, 2014; for in vivo imaging of dendritic spines, see Qiao et al., Cell Reports, 2022; for slice electrophysiology, see Chokshi et al., Neuron, 2019).
Data exclusions	No data were excluded from analyses.
Replication	Reported Ns for each experiment reflect total number of biological replicates. Each experiment contains a minimum of two independent experiments. Results were consistent across separate experiments and no data were excluded from analyses.
Randomization	Mice were randomly assigned to cages upon weaning. All mice underwent the same experimental procedures.
Blinding	Experimenters were blinded to genotypes throughout data acquisition and analysis.

Reporting for specific materials, systems and methods

We require information from authors about some types of materials, experimental systems and methods used in many studies. Here, indicate whether each material, system or method listed is relevant to your study. If you are not sure if a list item applies to your research, read the appropriate section before selecting a response.

Materials & experimental systems

n/a	Involved in the study
☐	☒ Antibodies
☒	☐ Eukaryotic cell lines
☒	☐ Palaeontology and archaeology
☐	☒ Animals and other organisms
☒	☐ Clinical data
☒	☐ Dual use research of concern
☒	☐ Plants

Methods

n/a	Involved in the study
☒	☐ ChIP-seq
☒	☐ Flow cytometry
☒	☐ MRI-based neuroimaging

Antibodies

Antibodies used

Antibody Source Identifier Concentration
Rabbit anti-ASPA (clone N1C3-2) GeneTex Cat# GTX113389; RRID AB_2036283 1:1000
Chicken anti-GFP Rockland Cat# 600-901-215; RRID AB_1537403 1:1000
Rat anti-MBP Millipore (clone 12) Cat# MAB386; RRID AB_94975 1:200
Rabbit anti-PDGFRα W.B. Stallcup N/A 1:200
Rabbit anti-cleaved Caspase3 (Asp175) Cell Signaling Cat# 9661S; RRID AB_2341188 1:200
Mouse anti-GFAP (clone GA5) Millipore Cat# MAB360; RRID AB_11212597 1:1000
Human anti-Sox9 R&D Systems Cat# AF3075; RRID AB_2194160 1:2000
Rabbit anti-Iba1 Wako Cat# 019-19741; RRID AB_839504 1:1000
Mouse anti-NF-L Degenotag Encor Cat# MCA-1D44; RRID AB_2923483 1:1000
Rabbit anti-NF-H Abcam Cat# ab8135; RRID AB_306298 1:1000
Mouse anti-PV (clone 235) Swant Cat# 235; RRID AB_10000343 1:1000
Biotinylated WFA Vector Labs Cat# B-1355; RRID AB_2336874 1:400
Rabbit anti-Caspr Peles lab N/A 1:600
Mouse anti-Bcas1 (5) Santa Cruz Cat# SC-136342; RRID AB_10839529 1:300
Goat anti-rabbit AlexaFluor 488 Thermo Fisher Scientific Cat# A-11034; RRID:AB_2576217 1:1000
Goat anti-rabbit AlexaFluor 594 Thermo Fisher Scientific Cat# A-11012; RRID:AB_2534079 1:1000
Goat anti-rabbit AlexaFluor 647 Thermo Fisher Scientific Cat # A-21245; RRID:AB_2535813 1:1000
Goat anti-chicken AlexaFluor 488 Thermo Fisher Scientific Cat # A-11039; RRID:AB_142924 1:1000
Goat anti-rat AlexaFluor 647 Thermo Fisher Scientific Cat# A-21247; RRID:AB_141778 1:1000
Goat anti-rat AlexaFluor 488 Jackson ImmunoResearch Cat# 112-545-167; RRID:AB_2338362 1:1000
Goat anti-mouse AlexaFluor 488 Jackson ImmunoResearch Cat# 115-545-166; RRID:AB_2338852 1:1000
Goat anti-mouse AlexaFluor 647 Thermo Fisher Scientific Cat# A-21236; RRID:AB_2535805 1:1000
Goat anti-human AlexaFluor 594 Thermo Fisher Scientific Cat# A-11014; RRID:AB_2534081 1:1000
Alexa Fluor® 594 Streptavidin Jackson ImmunoResearch Cat# 016-580-084; RRID:AB_2337250 1:1000

Validation

Primary antibodies used in this study were previously validated by manufacturers for specificity and/or were previously published by our lab and others. Rabbit anti-ASPA antibody validation: positive control - ASPA-transfected 293T (performed by manufacturer); also see Xin et al., Cell Reports 2019 and Larson et al., eLife 2018 for additional applications. Chicken anti-GFP antibody validation: negative control - absence of signal in GFP-negative tissue (performed in Chan lab). Rat anti-MBP antibody: see Haines et al. Nature Neuroscience 2015 and Lodato et al., Nature Neuroscience 2014 for additional applications. Rabbit anti-PDGFRα validation: negative control - knockout tissue (performed in Chan lab); see Mayoral et al., Cell Reports 2018 for additional applications. Rabbit anti-cleaved Caspase3 antibody: used in over 9000 citations; see Bhadury et al., Oncogenesis 2013 and Sun et al., Nature Communications 2023 for additional applications. Mouse anti-GFAP antibody: manufacturer's statement "Anti-Glial Fibrillary Acidic Protein Antibody, clone GA5 detects level of Glial Fibrillary Acidic Protein & has been published & validated for use in IC, IH, IH(P) & WB with more than 65 product citations". Human anti-Sox9 antibody: see O'Shea et al., Nature Communications 2022 and Huang et al., Neuron 2020 for additional applications. Rabbit anti-Iba1 antibody: has been used in over 4000 citations; see Monai et al., Nature Communications 2016 and Noguchi et al., Communications Biology 2024 for additional applications. Mouse anti-NF-L Degenotag antibody: see Shaw et al., Brain Communications 2023 for validation by manufacturer. Rabbit anti-NF-H antibody: see Kodati et al, Biomedicines 2022 and Kulesskaya et al., Frontiers in Cell and Developmental Biology 2022 for additional applications. Mouse anti-PV antibody: see Hoseini et al., eLife 2021 and Berdugo Vega et al., Nature Communications 2020 for additional applications. Biotinylated WFA antibody: see Okur et al., Nature 2024 and Lupori et al., Cell Reports 2023 for additional applications. Rabbit anti-Caspr antibody: previously validated in knockout tissue (performed in Peles and Chan labs); see Chang et al., eLife 2021 and Eshed-Eisenbach et al., Neuron 2020 for additional applications. Mouse anti-Bcas1 antibody: see Cunha et al., Journal of Experimental Medicine 2020 and Maas et al., Nature Communications 2020 for additional applications.

Animals and other research organisms

Policy information about studies involving animals; ARRIVE guidelines recommended for reporting animal research, and Sex and Gender in Research

Laboratory animals

NG2CreER:tau- mGFP mice (Zhu et al. 2011, Jax # 008538; Hippenmeyer et al. 2005, Jax # 021162), aged P26-35
PDGFRαCreER:MyrfFl/Fl mice (Kang et al. 2010, Jax # 018280; Emery et al. 2009, Jax # 010607), aged P21-90
Thy1-YFP-H mice (Feng et al., 2000, Jax # 003782), aged P60-90

Wild animals

No wild animals were used in this study.

Reporting on sex	Both male and female mice were used in this study. Due to the challenge of obtaining sufficient numbers of mice with the appropriate genetic crosses, groups were not balanced by sex.
Field-collected samples	No field-collected samples were used in this study.
Ethics oversight	All mice were handled in accordance with, and all procedures approved by, the Institutional Animal Care and Use Committee of the University of California San Francisco.

Note that full information on the approval of the study protocol must also be provided in the manuscript.

Plants

Seed stocks	N/A
Novel plant genotypes	N/A
Authentication	N/A
